# Smoking and HIV: time for a change?

**DOI:** 10.1186/1741-7015-11-16

**Published:** 2013-01-22

**Authors:** Nicola Petrosillo, Stefania Cicalini

**Affiliations:** 1 National Institute for Infectious Diseases "L. Spallanzani", Via Portuense 292, Rome, Italy

**Keywords:** HIV, smoking, mortality, cardiovascular, pulmonary, tuberculosis, cancer, opportunistic infections

## Abstract

Cigarette smoking is one of the most important causes of morbidity and mortality in the general population, and is a well-recognized risk factor for a variety of serious clinical conditions, including cardiovascular diseases, pulmonary diseases and cancers.

Smoking-related morbidity and mortality are of particular concern in patients with HIV infection, as the prevalence of current cigarette smoking is higher among HIV-positive patients than among the general population.

In a study by De *et al*., it has been evidenced that smoking is a risk factor for bacterial pneumonia in HIV-positive patients and smoking cessation reduces this risk.

HIV-positive patients who smoke have significantly increased mortality compared to those who have never smoked, indicating that smoking confers different mortality risk in HIV-positive as compared to HIV-negative patients, and lifestyle-related factors may pose a greater hazard to long-term survival of HIV-positive patients than those related to the HIV infection *per se*.

The high prevalence of smoking among HIV population, the many health risks that can result from this behavior, and the proven efficacy of cessation interventions in HIV-positive patients should encourage HIV care providers to make smoking cessation a high priority.

## Background

Cigarette smoking is a leading cause of morbidity and mortality in the general population [1], and it acts as a risk factor for many serious clinical conditions, including coronary artery disease, myocardial infarction (MI), stroke and pulmonary diseases. Moreover, in the general population, smoking increases the risk for many types of cancers, including cancers of the oral cavity, pharynx, esophagus, stomach, pancreas, larynx, lung, cervix, urinary bladder and kidney [2].

The prevalence of cigarette smoking is higher in HIV-positive patients as compared to the general population, with many studies reporting rates over 40% [3,4]. This raises concern on a possible relationship between smoking and the increased rate of co-morbidities and deaths due to cardiovascular (CV) disease and cancer observed among HIV-positive patients.

In a research article, De *et al*. addressed an important issue, namely the influence of smoking on pneumonia in HIV-positive patients, and evidenced that smoking cessation can reduce the risk for bacterial pneumonia by about 27%.

## Smoking as a risk factor for AIDS- and non-AIDS diseases

With the introduction of combination antiretroviral therapy (ART), which resulted in a reduction of AIDS -related diseases and deaths, serious non-AIDS diseases, including CV diseases, pulmonary diseases and cancers have become more common in HIV-positive patients. The prevalence of these co-morbidities is higher among HIV-positive patients than among individuals of the same age in the background population [5], suggesting a synergistic effect of underlying behavioral and pathological conditions.

Not unexpected, smoking may even further increase the risk for occurrence of these diseases (Table 1); for example, in the Data Collection on Adverse Events of Anti-HIV Drugs Study Group, the largest observational study of CV risk on HIV-positive on ART that demonstrated an association between the risk of MI and combination ART, particularly protease inhibitor therapy, it has been shown that the adjusted relative rate of MI per years of exposure to protease inhibitors was 1.16, compared to 2.83 for current smoking [6].

**Table 1:** Clinical conditions influenced by smoking in HIV-positive patients

Cardiovascular disease	-Coronary artery disease -Myocardial infarction -Stroke
Pulmonary hypertension	
Pulmonary disease	-Chronic obstructive pulmonary disease -Bacterial pneumonia -Pulmonary tuberculosis
Cancers	
Opportunistic infections	-Oral candidiasis -Oesophageal candidiasis -*Pneumocystis *pneumonia
Bone disease and fractures	

More recently, in the Strategies for Management of Antiretroviral Therapy (SMART) study, HIV-positive current smokers had a two-fold risk of major cardiovascular events as compared to non-smokers [3].

Respiratory complications of smoking include chronic obstructive pulmonary disease (COPD) and respiratory infections, such as bacterial pneumonia and pulmonary tuberculosis (TB), and HIV infection may increase susceptibility to both of these [7,8].

Cigarette smoking is a major trigger for COPD in the general population as well as in the HIV population, and studies of HIV-positive patients have identified a clear association between bacterial pneumonia and smoking, with an over two-fold risk of bacterial pneumonia in HIV-positive current smokers as compared to non-smokers [3].

In a research article published in *BMC Medicine*, Preeti De and colleagues conducted a systematic review and meta-analysis of cohort and case-control studies to assess whether smoking cessation reduces the incidence of both bacterial pneumonia and *Pneumocystis *pneumonia in HIV-positive patients. The authors provide evidence that HIV-positive current smokers are at increased risk for bacterial pneumonia than former smokers. Moreover, there is no evidence that HIV-positive former smokers were at higher risk than never smokers, indicating that smoking cessation reduces this risk. Thus, smoking cessation programs should be systematically integrated into HIV care.

However, further studies are needed to investigate how long the risk of current smoking remains elevated after stopping before the benefits of cessation become manifest.

There is increasing evidence that smoking may also have a major direct impact on pulmonary vasculature and studies suggested that tobacco smoke causes a pulmonary vascular remodelling that resembles that occurring in patients with pulmonary hypertension, a rare but severe manifestation in the course of HIV disease, leading to right ventricular failure and, ultimately, death [9].

Smoking has also been demonstrated to be a significant risk factor for TB. Recent meta-analyses indicated that in the general population smokers are almost twice as likely to be infected with TB (relative risk (RR) around 1.5 for latent TB infection) and to progress to active disease (RR around 2.0 for TB disease) [10-12].

The risk of developing TB is over 20 times greater in HIV-positive patients than among those who do not have HIV infection. If smoking increases the impact of TB in HIV-negative patients, its effect in HIV-positive patients may be significantly greater, with a deleterious synergistic interaction between smoking, HIV and TB [8].

HIV-positive patients who smoke have an increased risk of a variety of opportunistic infections, including oral and esophageal candidiasis [3,13]. Some studies have also identified smoking as a risk factor for *Pneumocystis *pneumonia [14], even though this association has not been evidenced in the study by De *et al*.

A number of malignancies have also been associated with HIV infection. For example, HIV-positive patients are at increased risk for many cancers associated with human papillomavirus, including cancer of the cervix, anus, vulva, vagina, penis, oral cavity and pharynx [15]. However, previous findings of an association between cigarette smoking and abnormal anal cytological abnormalities among both HIV-positive women and men, have not been more recently confirmed [16].

Lung cancer is the most important cause of non-AIDS-defining malignancy in HIV-positive patients. Although a number of studies have demonstrated that HIV infection is associated with a significantly increased risk for developing lung cancer, independently of smoking status [17], smoking may additionally increase lung cancer risk. For HIV-positive patients in the SMART study, the adjusted hazard ratio for lung cancer for current versus never smokers was 9.4 [3].

HIV-positive patients may be at greater risk for metabolic bone disease, including osteopenia and osteoporosis [18]. Smoking has been associated with an increased risk of loss of bone density, and is an independent risk factor for fractures among HIV-positive patients [19].

## Smoking and survival in HIV-positive patients

HIV-positive patients who smoke have significantly increased mortality compared to those who have never smoked [3,4]. In a US veterans study, the mortality rate per 100 person-years was 1.76 for HIV-negative never smokers, 2.45 for HIV-positive never smokers and 5.48 for HIV-positive current smokers, suggesting that both HIV infection and smoking contribute to increased mortality compared to never smokers [4].

In a recently published article from the Danish HIV Cohort study, it has been observed that the excess mortality of smokers is tripled and the population-attributable risk of death associated with smoking is doubled among HIV-positive patients compared to the background population [20]. These data indicate that smoking confers different mortality risk in HIV-positive than in HIV-negative patients, and lifestyle-related factors may pose a greater hazard to long-term survival of HIV-positive patients than those related to the HIV infection itself.

Moreover, mortality was lower among previous compared to current smokers and this emphasizes the importance of counselling HIV-positive patients on smoking cessation, as smoking may impact their life expectancy considerably more than HIV infection *per se *[20].

## Smoking cessation as a prevention tool

Although further studies are needed to elucidate whether smoking has an additive or synergistic effect on cardiovascular, pulmonary and neoplastic diseases in HIV-positive patients, without doubt smoking is the preventable factor that accounts for the highest number of life-years lost in the general population and, particularly, in the HIV population.

However, although large clinical evidence exists to support the use of a variety of smoking cessation interventions in the general population, there are no clinical practice guidelines to guide the provision of smoking cessation treatment in HIV-positive patients.

The high prevalence of smoking among the HIV population, the many health risks that can result from it, and the proven efficacy of cessation interventions in HIV-positive patients should encourage clinicians to make smoking cessation for their patients a high priority.

HIV-positive patients should be screened for smoking, and current smokers should be enrolled in smoking cessation programs as a part of care in HIV-positive patients.

HIV-positive smokers need to be informed about the impact of smoking on HIV disease and its treatment. In a setting where HIV care is well organized and ART is available free of charge, HIV-positive smokers should be aware that they lose more life-years to smoking than to HIV infection [20].

## Conclusions

The bulk of evidence supports the need for smoking cessation in HIV-positive patients. Life quality and expectancy could improve as a consequence of this behavioral change.

In the article by De *et al*., it has been shown that smoking cessation is causally associated with a reduced risk of bacterial pneumonia in HIV-positive patients. Therefore, all the efforts of clinicians and other HIV-care providers should be addressed to prevent all the co-factors that increase the risk for AIDS- and non-AIDS-related diseases.

## Abbreviations

ART: antiretroviral therapy; COPD: chronic obstructive pulmonary disease; CV: cardiovascular; MI: myocardial infarction; RR: relative risk; SMART study: Strategies for Management of Antiretroviral Therapy; TB: tuberculosis

## Competing interests

Dr. Petrosillo received honorary fees as a speaker for GSK, Gilead, Pfizer, MSD, Johnson & Johnson, Carefusion, Novartis, Sanofi Aventis, Janssen Cilag, Astellas and as a member of scientific board for Janssen Cilag, Carefusion, Pfizer and MSD. Dr. Cicalini has no competing interest to declare.

## Authors' contributions

NP and SC each contributed drafts of the original manuscript. Both authors read and approved the final version of the manuscript to be published.

## Authors' information

Drs Petrosillo and Cicalini are at the National Institute of Infectious Diseases "Lazzaro Spallanzani", Rome, Italy.

## Pre-publication history

The pre-publication history for this paper can be accessed here:

http://www.biomedcentral.com/1741-7015/11/16/prepub
